# The effect of forward postural lean on running economy, kinematics, and muscle activation

**DOI:** 10.1371/journal.pone.0302249

**Published:** 2024-05-29

**Authors:** Nina M. Carson, Daniel H. Aslan, Justus D. Ortega

**Affiliations:** 1 School of Applied Health, California State Polytechnic University Humboldt, Arcata, CA, United States of America; 2 Department of Biological Sciences, University of Southern California, Los Angeles, CA, United States of America; Ningbo University, CHINA

## Abstract

**Background:**

Running economy, commonly defined as the metabolic energy demand for a given submaximal running speed, is strongly associated with distance running performance. It is commonly believed among running coaches and runners that running with increased forward postural lean either from the ankle or waist improves running economy. However, recent biomechanical research suggests using a large forward postural lean during running may impair running economy due to increased demand on the leg muscles.

**Purpose:**

This study tests the effect of altering forward postural lean and lean strategy on running economy, kinematics, and muscle activity.

**Methods:**

16 healthy young adult runners (23±5 years, 8M/8F) ran on a motorized treadmill at 3.58m/s using three postural lean angles [upright, moderate lean (50% of maximal lean angle), and maximal lean] and two strategies (lean from ankle and lean from waist [trunk lean]). Metabolic energy consumption, leg kinematics, and muscle activation data were recorded for all trials.

**Results:**

Regardless of lean strategy, running with an increased forward postural lean (up to 8±2 degrees) increased metabolic cost (worsened economy) by 8% (*p* < .001), increased hip flexion (*p* < .001), and increased gluteus maximus (*p* = .016) and biceps femoris (*p* = .02) muscle activation during the stance phase. This relation between running economy and postural lean angle was similar between the ankle and trunk lean strategies (p = .743).

**Conclusion:**

Running with a large forward postural lean reduced running economy and increased reliance on less efficient extensor leg muscles. In contrast, running with a more upright or moderate forward postural lean may be more energetically optimal, and lead to improved running performance.

## Introduction

The popularity for running as a form of exercise, recreation and competition has grown significantly over the last quarter century [1]. A major concern for modern runners is how to improve performance. To improve running performance, many runners and coaches seek out ways to optimize running technique. However, modifications to running technique are not always backed by scientific evidence. A recurrent claim is that running with a forward lean can improve running performance [2, 3]. Understanding how physiological and biomechanical outcomes of running change as forward lean angle increases could provide valuable insight to assist coaches and runners in implementing science-based running techniques to improve running performance. Moreover, running with a forward lean can be accomplished by using different postural strategies, (e.g., leaning from the ankle or leaning from the waist), and it is unknown if different postural lean strategies can influence biomechanical and physiological outcomes. Here we will examine how running with varying degrees of forward lean angles, accomplished using different lean strategies, affects running economy, muscle activation, and kinematic outcomes.

Running economy (the rate of steady-state oxygen consumption and carbon dioxide production at specific running speeds) [4] is considered a primary determinant of endurance running performance [5–9]. The most influential contributors to the metabolic cost of running (which directly corresponds to running economy) are the cost of generating muscle force to support body weight during stance phase [10, 11], horizontal propulsion [12], and the cost to swing the leg [13] respectively [14]. These major determinants of running economy can be influenced by alterations in running technique, such as forward postural lean, by changing kinematics and muscle activity [15–17].

The current evidence regarding the effect of forward lean on running economy and muscle activation is limited. Williams and Cavanagh [18] found that the most optimal running economy was observed when runners used a mean trunk flexion angle of 5.9 degrees, as opposed to more upright postures. However, this study did not control forward lean while running, but instead examined the natural trunk lean in runners classified from good to poor economy. Furthermore, there has not been any studies that have looked at if forward lean while running influences muscle activation patterns. However, walking studies that have systematically increased flexion of the trunk, hip, and knee have shown increased extensor muscle activation resulting in greater metabolic cost of walking (worse economy) [19, 20]. Foster et al., [21] demonstrated that walking with more flexed joints changes the ratio between the muscle moment arm to the ground reaction moment arm (known as the effective mechanical advantage [EMA]), resulting in worse EMA compared to upright walking. Using inverse dynamics, they identified this reduced EMA is what increased knee extensor muscle activation which drove the higher metabolic cost of walking in these flexed postures. Although walking is characterized differently than running, these principles of physics may be similar in both gaits.

Leaning forward at the trunk while running has been shown to alter kinetics and kinematics in ways that may increase extensor muscle activation and impair optimal running economy, but results are also limited. Teng and Powers [22] observed that running with a high degree of trunk flexion (~11 degrees) caused a 140% increase in positive hip work [23]. Similarly, Warrener et al., [24] observed that greater trunk flexion while running was associated with increased hip extensor moments. This could be due to flexed joints creating greater external flexion moments that need to be counteracted by extensor muscle force (worse EMA). To produce this muscle force, increased muscle activation is required, which could lead to a higher metabolic cost of running. Together, these studies suggest that running with a flexed trunk may require increased hip extensor muscle activation that ultimately leads to decreased running economy. However, although running with an increased forward trunk lean may require increased hip extensor muscle activation, Teng and Powers [25] also found that this running posture reduces knee extensor work and may reduce patellofemoral joint stress. While Warrener et al., [24] found that this increased trunk flexion coincided with higher vertical ground reaction force impact transient peaks (at the moment of impact) and an elevated rate of loading (the slope of the vertical ground reaction force), they also showed knee extensor moments decreased by 22% with greater trunk flexion. Interpreting the pros and cons of running with a forward postural lean from these results are complex and limited, and these results provide sparse insight into how forward trunk lean may influence running economy and muscle activation patterns. Further highlighting the importance of comprehensively understanding how running posture influences running economy, muscle activation, and kinematics.

Additionally, while some researchers have specifically looked at running with a forward lean at the trunk (trunk flexion), there is even less research on how running with other lean strategies to achieve a forward body lean (i.e., ankle strategy) influences running biomechanics and performance. As such, the effects of running with a forward body lean on running economy, muscle activity, and kinematics are not well understood. If coaches aim to prescribe running techniques to optimize performance by improving running economy and muscle activation patterns, further research is essential to fully understand these complex relationships.

This study aimed to investigate the effect of postural lean (lean strategy and forward body lean angle) on running economy, kinematics, and muscle activation. While some prior literature may suggest that increasing forward postural lean angle may reduce running economy, the body of evidence is minimal and the mechanism for this increased cost is only speculated. Thus, based on existing but limited literature, this study tests the null hypotheses that running economy, kinematics, and muscle activation patterns would be similar across a range of forward postural lean angles (upright, moderate, and maximal forward lean) and between lean strategies (ankle versus torso lean).

## Materials and methods

### Participants

Sixteen healthy young adult runners (23 ± 5 years, 8 women and 8 men) participated in our study. Participant recruitment took place between May 2015 and February 2016. Study participants included adults who 1) run for fitness or competition, with a 5k time ≤ 22 minutes and 2) were without any cardiovascular, neurological, or orthopedic disorders in the 6 months prior to participation. Before the experiment, all participants gave their written informed consent. No minors or other vulnerable populations participated in this study. This protocol was approved by the California State Polytechnic University, Humboldt Institutional Review Board (IRB # IRB 14–217). To estimate sample size, we performed a power analysis using metabolic data from a prior study examining the relation between metabolic cost (economy) and trunk angle [18]. We calculated a sample size of 13 subjects was needed to achieve a large Cohen’s d effect size of 1.047 with a power of 0.8 for detecting significant differences (alpha = 0.05). We chose a sample size of 16 to ensure we will have sufficient power for detecting differences (alpha = 0.05) across trials, conditions, and variables to provide a conservative sample if effect sizes are smaller in our study.

### Protocol

**Fig 1:**
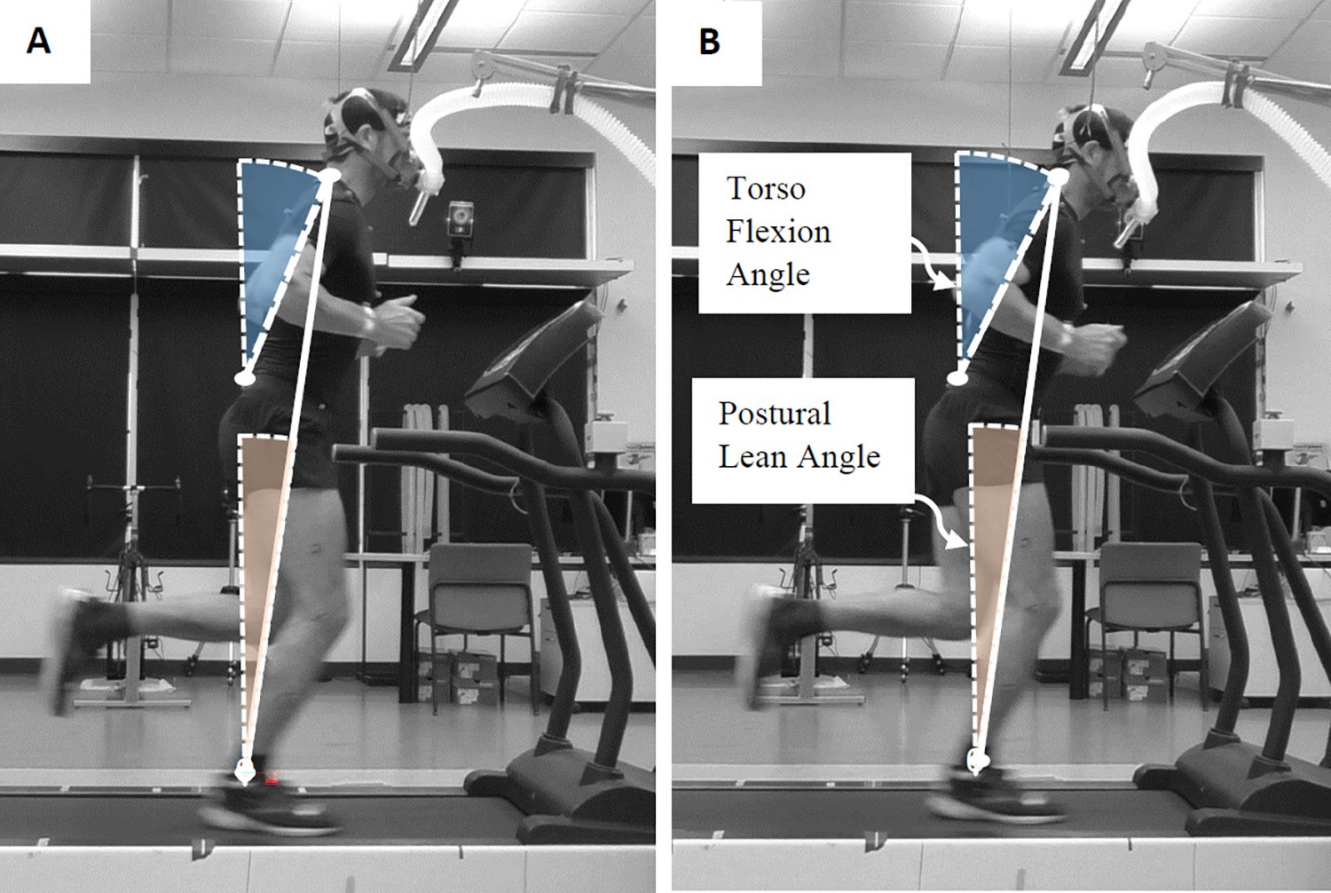
Image of running with ankle (A) and torso (B) postural lean strategy. Solid line represents total postural lean angle and hatched (—-) line represents the torso flexion angle. Images shown here are similar to the sagittal view video feedback used for the prescribed moderate and maximal lean trials. Participants were asked to maintain the prescribed postural lean angle throughout the trial using the prescribed lean strategy (ankle or torso).

Each participant completed one experimental session. Initially, participants were familiarized to treadmill running for 5 minutes at 3.58 m/s (8 mph) on a level, motorized treadmill (Trackmaster TMX425C, Full Vision Inc., Newton, KS, USA). Following treadmill familiarization, participants performed a total of five experimental running trials: upright (minimal lean), moderate postural lean from the ankle, maximal postural lean from the ankle, moderate postural lean from the torso, and maximal postural lean from the torso (Table 1; Fig 1). For each trial, participants ran for 5 minutes at the intermediate speed of 3.58 m/s (8 mph) and were given a minimum of 5 minutes rest between trials to minimize the influence of fatigue or excess post-exercise oxygen consumption (EPOC) on subsequent trials. For the *ankle strategy* trials, subjects were instructed to “lean forward from the ankle” and for the *torso strategy* trials, subjects were instructed to “lean from the hip”. To help minimize a trial order effect we used a semi-randomized counterbalanced trial order. Specifically, each participant started by running with either an upright posture or running with a maximal forward lean from the ankle. For the upright trial, participants were instructed to “run as upright as possible minimizing the lean forward”. For the maximal forward lean trials, participants were instructed to “run with as far forward of a lean as you can while leaning from the ankle”. Using kinematic data from the maximal forward lean trial, we calculated each participant’s maximal postural lean angle and then used this to identify the prescribed maximum and moderate (50% of max lean) lean angle used in the remaining trials. Following the randomly ordered upright and maximal forward lean (ankle strategy) trials, participants then ran with the prescribed moderate forward lean from the ankle (ankle strategy) using real-time video biofeedback to achieve the prescribed lean angle. Only occasionally in the first minute of a trial, research staff would need to remind the subject to “try your best to match the prescribed lean angle on the video monitor”. Participants then repeated the maximal forward lean and moderate forward lean angle trials, in random order, while being instructed to “lean from the hip and use the video feedback to match the prescribed (total) postural lean angle”.

**Table 1:** Metabolic and kinematics data for each postural lean condition (N = 16).

		Postural Lean				
		Moderate			Large	
	Upright	Ankle	Torso		Ankle	Torso
Postural Lean Angle (degrees)	1.7 ± 0.7	4.3 ± 0.8	4.3 ± 1.0		8.2 ± 1.4	8.4 ± 1.9^†^
Torso Flexion Angle (degrees)	4.9± 1.0	12.1 ± 2.5	16.3 ± 2.3*		17.4 ± 2.8	21.8 ± 4.2*^,†^
Net Metabolic Power (W·kg^-1^)	11.54 ± 1.17	11.69 ± 1.32	11.8 0 ± 1.22		12.31 ± 1.58	12.20 ± 1.74^†^
Ground Contact Time (ms)	252 ± 8	253 ± 9	256 ± 8		255 ± 8	255 ± 8
Leg Swing Time (ms)	428 ± 12	429 ± 15	427 ± 13		443 ± 15	427 ± 12
Stride Length (m)	2.43 ± .04	2.44 ± .04	2.44 ± .04		2.49 ±. 05	2.43 ± .04
Peak Hip Flexion (degrees)	28.6 ± 1.4	31.6 ± 1.5	33.1 ± 1.9		37.6 ± 1.3	35.4 ± 1.9^†^
Peak Knee Flexion (degrees)	40.6 ± 5.5	40.7 ± 1.4	41.8 ± 1.4		42.2 ± 1.6	41.0 ± 1.5^†^
Peak Ankle Dorsiflexion (degrees)	22.0 ± 4.1	24.6 ± 0.9	25.1 ± 1.1		25.3 ± 1.1	25.0 ± 1.1

Data are presented as Mean ± SD. Asterix (*) indicate significant difference between Ankle and Torso strategy (p < .01). Dagger (^†^) indicate significant effect of postural lean angle across all postural lean angle conditions and both lean strategy conditions (p < .01).

To help participants maintain each prescribed forward lean angle and lean strategy, we provided real-time 2-D sagittal (side view) video feedback (60 Hz) of the participant’s running posture throughout each running trial. Specifically, on a monitor 1 meter in front of the participant, we projected the prescribed postural lean angle along with the real-time video of their postural lean. For each trial, participants aligned their body (i.e. postural lean angle) with the prescribed postural lean angle shown from a sagittal view on the video monitor.

### Data analysis

#### Running economy

For each of the five trials, running economy was quantified as metabolic power (W·kg^-1^) using indirect calorimetry (TrueOne 2400; Parvo Medics, Sandy, UT, USA). Average metabolic rate per kilogram body mass (W·kg^-1^) was calculated using the average $\dot{V}O_{2}$ (ml O_2_ min^-1^) and $\dot{V}CO_{2}$ (ml CO_2_ min^-1^) for two minutes between minutes 3 and 5 when the real-time plot of $\dot{V}O_{2}$ indicates that metabolic steady-state had been achieved [26]. Additionally, we monitored the respiratory exchange ratio (RER) throughout each trial to ensure that it remained below 1.0, indicating that oxidative metabolism was the main metabolic pathway. The standing metabolic rate was subtracted from running gross metabolic rate to calculate net metabolic power (W·kg^-1^) for each running trial.

#### Kinematics

For all running trials, we determined stride kinematics and postural lean angle using a nine-camera digital motion capture system (Frame rate = 200 Hz; Vantage+, Vicon Inc., Centennial, CO, USA) for 20 strides during the last two minutes of each trial. A set of 39 passive reflective markers were attached to the participant’s upper and lower body in accordance with Vicon Plug-In-Gait Full-body model for motion analysis [27]. The marker data were low-pass filtered at 6 Hz to reduce motion artifact (fourth-order Butterworth filter) and used to calculate spatiotemporal stride kinematics (stride length, time of foot-ground contact), as well as average and peak ankle, knee, and hip motion across the stance phase, swing phase, and entire gait cycle. Marker data was also used to calculate the average postural lean angle (absolute angle between a virtual segment from the stance ankle joint to c7 marker ball relative to the vertical axis) and torso flexion angle (sacral marker to c7 marker relative to the vertical axis) across the gait cycle.

#### Muscle activation

To determine lower limb muscle activation, we collected electromyographic (EMG) data from seven muscles of the right leg synchronously with our kinematic data, through the Vicon Motion capture system. After preparing the shaved skin with an impedance-lowering abrasive gel (NuPrep, Aurora, CO, USA), we placed differential electrodes with wireless pre-amplifiers (Trigno, Delsys Incorporated, Boston, MA, USA) over the following muscles of each participant’s right leg: gluteus maximus (GM), biceps femoris (BF), rectus femoris (RF), vastus medialis (VM), medial gastrocnemius (MG), soleus (SOL), and tibialis anterior (TA). We verified electrode positions and signal quality by visually inspecting the EMG signals, sampled at 2000 Hz, while participants contracted each muscle. We recorded and processed all EMG data for 20 strides during the last 2 minutes of each running trial and at the same time as kinematic data was collected. Prior to data analysis, EMG signals were band-pass filtered (20–450 Hz).

Using a custom Visual 3D analysis program (Germantown, MD, USA), we full wave rectified the raw EMG signals and calculated the normalized root mean square (40 ms window) EMG amplitude (EMG_RMS_). EMG_RMS_ amplitude of each muscle was normalized to the peak EMG_RMS_ amplitude during the upright running trial. We determined average EMG_RMS_ over the stance phase, swing phase, and entire stride, as well as over each 10% increment of the stride (0 to 10%, 10 to 20%, etc. 0% indicates initial heel strike).

### Statistics

To assess statistical differences in running economy and stride kinematics due to postural lean angle (upright, moderate, and large lean angle), postural lean strategy (ankle vs. torso), and determine any interaction of lean angle and lean strategy, we used linear mixed-effects models with participants being the random effect and postural lean angle and postural lean strategy being fixed effects. When a significant main effect of lean angle was observed, we performed post-hoc t-tests, with Bonferroni correction when appropriate, to determine differences in dependent variables between the three prescribed postural lean angles. In addition, we used Pearson R correlation analysis to determine the strength of relation between maximum lean and metabolic cost. We used mixed-effects models to assess statistical differences in muscle activation. Prior to all statistical analysis, we performed paired two-tailed t-tests to compare each biomechanical and metabolic variable from minutes 2.0 to 3.0 to the respective variable from minutes 3.5 to 5.0 to ensure participants achieved a biomechanical and metabolic steady-state. For all analyses, we set the level of significance at α = 0.05 and performed statistical analyses using SPSS version 29 software (IBM Corp., Armonk, NY, USA).

We ran follow-up statistical parametric mapping (SPM) sensitivity analyses on ankle, knee, and hip angles across ensembled traces for 20 strides normalized from zero to 100 of gait cycle for each condition of each subject. For these analyses we use a two-way factorial structure of the main effect of lean angle, postural strategy, and the interaction between the two to see if SPM{F} crosses a critical threshold at any normalized time point of the gait cycle (0–100%).

## Results

### Postural lean

For the initial maximal postural lean trial, participants were able to achieve an average maximal postural lean angle of 8.2° (range = 6.1–11.5°, SD 1.9°). Despite being asked to run with upright and near vertical posture, participants run with a modest forward postural lean of 1.7 ± 0.7° during the “upright” running trials. For the prescribed moderate postural lean trial using the ankle strategy, participants were able to maintain an average postural lean angle of 4.3 ± 0.8° and approximately 58% of the maximal lean angle. For the prescribed moderate and maximal lean condition using the torso lean strategy, participants achieved similar total postural lean angles as in the ankle lean strategy trials, *F*(1,61) = .24, *p* = .436 (Table 1); and for both lean strategies, total postural lean angle increased from the upright condition to the maximum lean angle condition, *F*(1,61) = 57.72, *p* < .001. When running using the torso strategy, participants used a 34% greater torso flexion angle as compared to running with the ankle strategy across both the moderate and maximal prescribed lean trials, *F*(1,61) = 33.80, *p* < .001, (Table 1).

### Running economy

In support of our first hypothesis, postural strategy (leaning from ankle vs. torso) had no main effect on the net metabolic cost of running across the range of postural lean conditions, *F*(1, 64) = 0.108, *p* = .743, and there was no significant interaction effect between postural lean angle and lean strategy on net metabolic cost, *F*(1, 64) = 1.343, *p* = .251.

**Fig 2:**
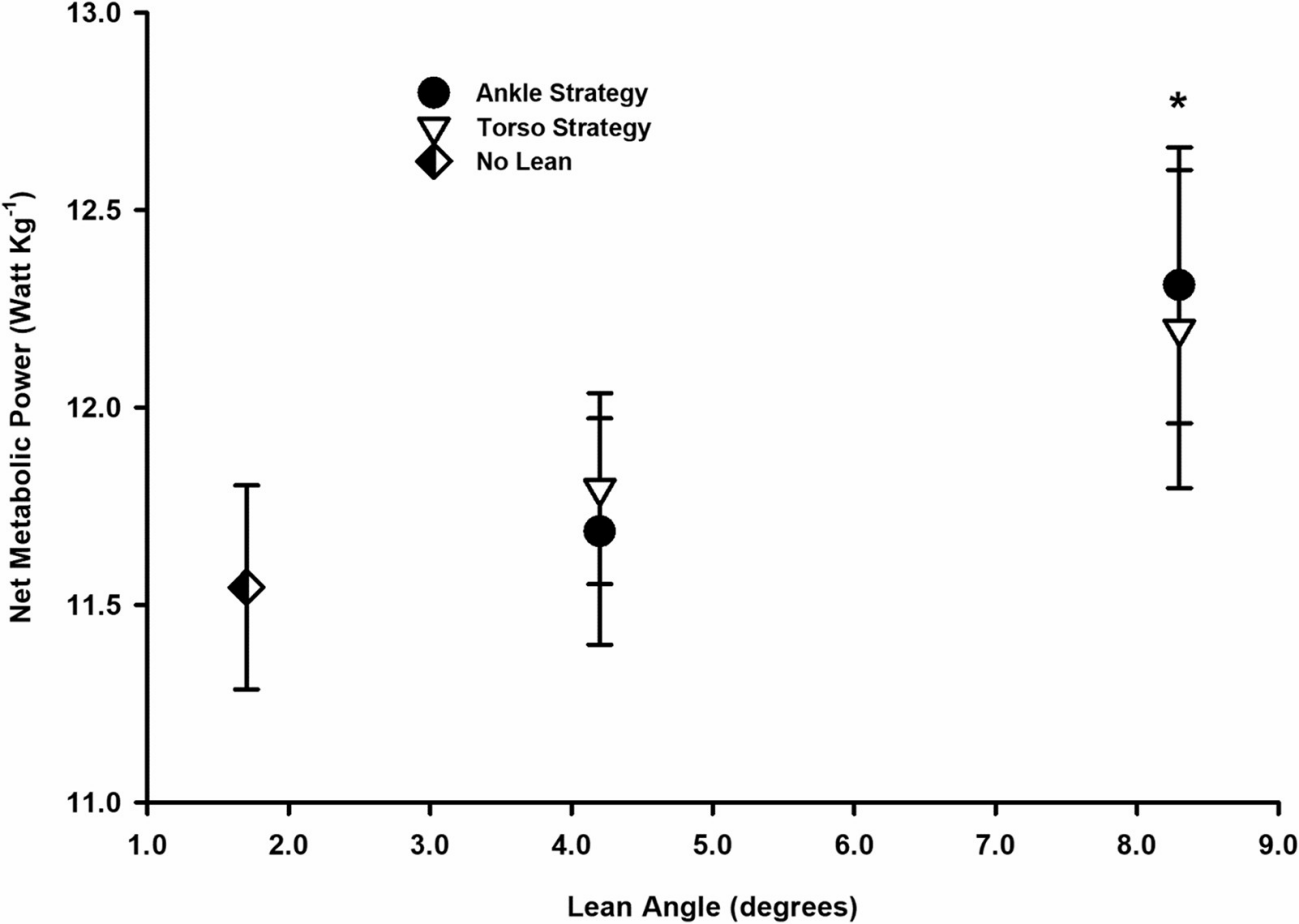
Mean (± SE) net metabolic power (Watt·kg^-1^) plotted as a function of postural lean angle for runners using no lean (⬖), ankle strategy (●), and torso strategy (▽). Asterisks (*) indicate significant differences from both no lean and moderate lean running (*p* < .05). Net metabolic cost increased with lean angle yet there was no significant difference in metabolic power between postural lean strategies (*p* = .700).

However, in contrast to our second hypothesis, increases in forward postural lean significantly increased net metabolic cost by 8% (worsened running economy) *F*(1, 64) = 11.592, *p* < .001 (Fig 2). Contrast revealed that a moderate postural lean angle increased net metabolic cost by ~2% compared to the upright lean angle, *F*(1, 15) = 8.00, *p* = .013, and a large postural lean angle increased net metabolic cost by ~ 6% compared to the moderate lean angle, *F*(1,15) = 8.48, *p* < .001. Moreover, participants that ran with a greater maximum lean angle exhibited a significantly greater net cost; a relation best expressed using a linear model (Y = 9.17+.56X; [r = .527, p = .002])

#### Kinematics

**Fig 3:**
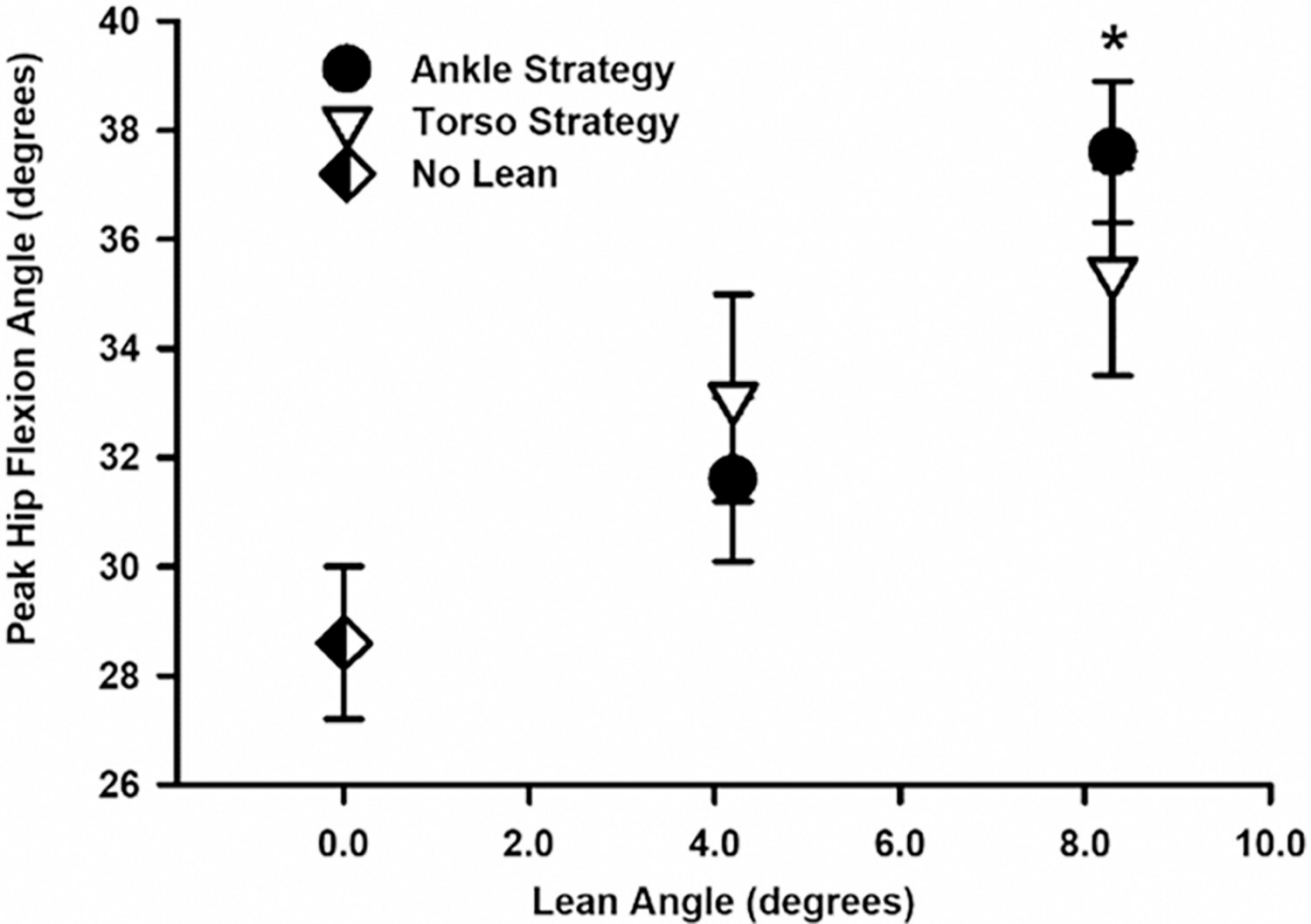
Mean (±SE) peak hip flexion angle (°) during the stance phase plotted as a function of postural lean angle for runners using no lean (⬖), ankle strategy (●) and torso strategy (▽). Asterisks (*) indicate significant differences from both no lean and moderate lean angles (*p* < .05). Peak hip flexion angle increased with lean angle yet there was no significant difference in peak hip flexion angle between postural lean strategies (*p* = .570).

In addition to changes in running economy, forward postural lean influenced stride kinematics. Peak hip flexion during the stance phase increased by 28% (~7 degrees) between the upright and large lean conditions, *F*(1, 64) = 23.831, *p* < .001 (Table 1; Fig 3). Contrast revealed that a moderate postural lean angle increased peak hip flexion angle by ~3 degrees compared to the upright lean angle, *F*(1, 15) = 44.77, *p* < .001, and a large postural lean angle increased peak hip flexion angle by ~ 4 degrees compared to the moderate lean angle, F(1, 15) = 39.94, *p* < .001.

While lean strategy had no significant main effect on peak hip flexion angle, *F*(1, 64) = .308, *p* = .580, there was a significant interaction effect between postural lean angle and lean strategy on peak hip flexion angle, *F*(1, 64) = 8.666, *p* = .005. As peak hip flexion angle increased with the greater lean angle in both strategy conditions, post-hoc analysis revealed that peak hip flexion angle increased more when using the ankle lean strategy (Table 1).

In the follow-up SPM sensitivity analysis, there was a significant main effect of postural lean angle on hip angle from 0–40% and 80–100% of the gait cycle SPM{F}(2,30) = 6.242, p < .001, p = .006; respectively. While there was no effect of lean strategy on SPM hip angle results, there was a significant interaction between postural lean angle and lean strategy at 5–20% and 85–90% of the gait cycle SPM{F}(2,30) = 6.242, p = .006, p = .047, respectively.

Similar to the effect on peak hip flexion, there was a significant effect of forward postural lean on peak knee flexion angle during the stance phase of running, *F*(1, 64) = 13.083, *p* < .001 (Table 1). Although significant, peak knee flexion angle only increased by ~2 degrees across all lean angle conditions. There was no significant main effect of lean strategy on peak knee flexion angle, *F*(1, 64) = .072, *p* = .789. However, there was a significant interaction effect between postural lean angle and lean strategy on peak knee flexion angle, *F*(1, 64) = 12.284, *p* < .001. This indicates that lean angle had different effects on peak knee flexion angle depending on which lean strategy was used. Specifically, while using the ankle strategy, peak knee flexion during stance did not significantly change between upright and moderate lean angles (p = .726), but increased between the moderate to large lean angle, *F*(1, 15) = 4.94, p = .032. When using the torso strategy, peak knee flexion angle increased minimally between upright and moderate lean angles, *F*(1,15) = 10.75, *p* = .005, but did not change significantly between the moderate to large lean angle (*p* = .108). It should be noted that while statistically significant, these changes in peak knee flexion angle due to changes in lean angle were all very small (~1–2 degrees). In the follow-up SPM sensitivity analysis, there was no main effect of postural lean angle, lean strategy, or the interaction between them on knee angle across the ensembled 0–100% gait cycles.

Despite the significant effect of postural lean angle on peak hip and knee flexion, there was no effect of postural lean angle on peak dorsiflexion during the stance phase (*p*>.05, Table 1). In evaluating whole-body spatiotemporal kinematics, we observed a significant interaction effect between lean strategy and postural lean angle, *F*(1, 64) = 7.242, *p* = .009. Specifically, stride length increased modestly (~5cm) from the upright to the large postural lean angle condition when running with an ankle lean strategy (*p* = .029) but did not change when running with a torso lean strategy (*p* = .812). Moreover, there was no main effect of postural lean angle or lean strategy (ankle vs. torso) or any interaction effect of lean angle and lean strategy on running step width, ground contact time, or swing time (*p*>.05). In the follow-up SPM sensitivity analysis, there was no main effect of postural lean angle, lean strategy or the interaction between them on ankle angle across the ensembled 0–100% of gait cycles.

### Muscle activity

**Fig 4:**
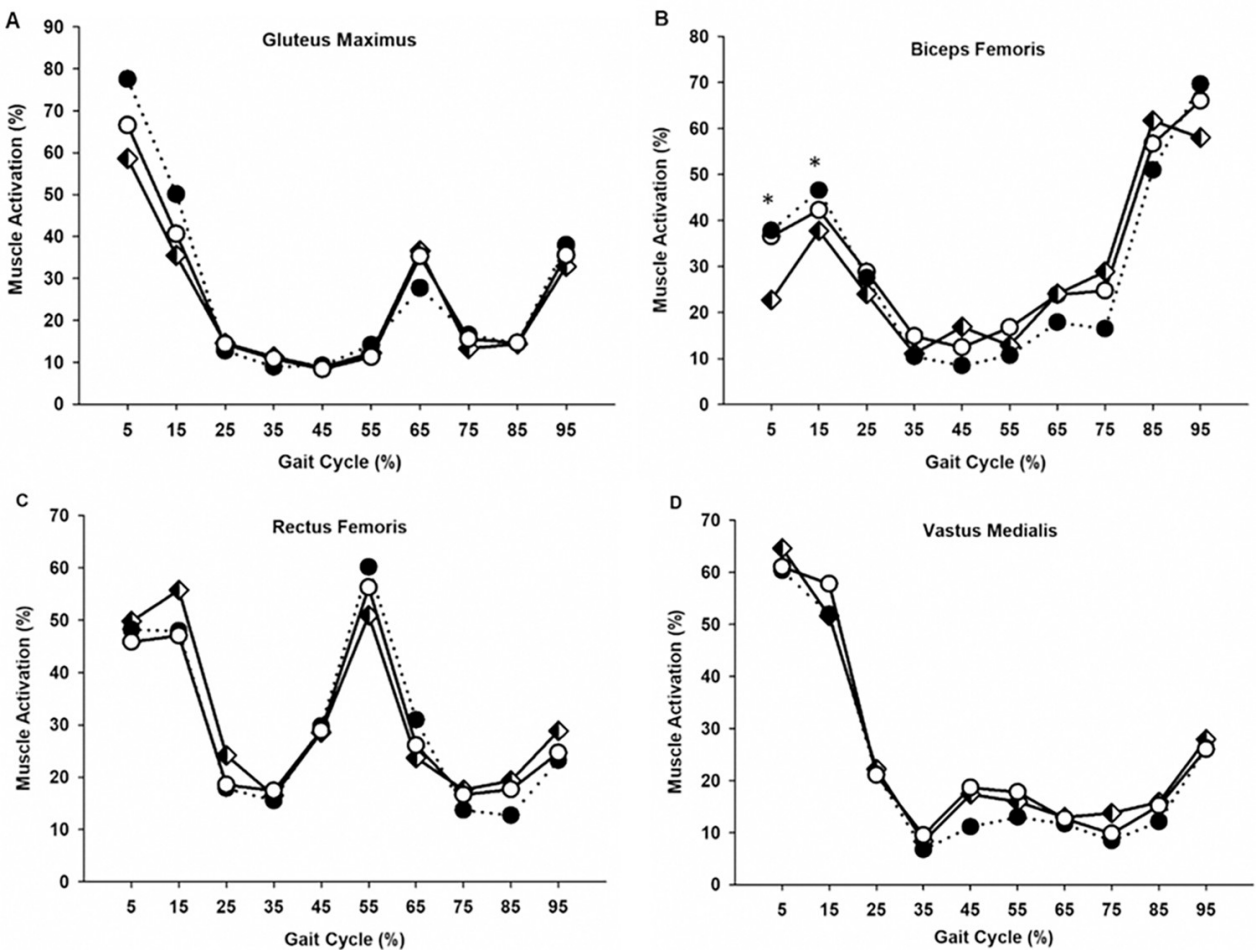
Average normalized EMG_RMS_ signals of the (A) gluteus maximus, (B) biceps femoris, (C) rectus femoris, and (D) vastus medialis muscles for the no lean (0 deg, ⬖), moderate lean (4.3 deg, ⬜), and large lean (8.3 deg, ⬤) conditions over a complete stride (0–100% of gait cycle). Each data point represents the average of ankle and torso lean strategies. Tibialis Anterior, Soleus, and Medial gastrocnemius activity exhibited no significant effects of lean angle or lean strategy, and thus are not shown. Asterisks (*) indicate significant differences from the large postural lean condition (p < .05).

To determine whether leg muscle activation was influenced by postural lean angle or lean strategy, we averaged EMG_RMS_ signals across stance and swing phases, and for every 10% of the gait cycle. While our analysis showed no significant effect of lean strategy on any muscle activation variable, it did reveal a significant main effect of postural lean angle on stance phase GM and BF muscle activation (*p* < .05, Table 2). For both the ankle and torso lean strategies, stance phase GM muscle activation increased by 31% between upright and the large lean condition *F*(1, 42) = 17.518, *p* < .001 (Table 2). Specifically, within the first 20% of the gait cycle (loading response), GM activation increased by 45–50% from the upright to large postural lean angle conditions [0–10%: *F*(2, 54) = 20.977, *p* < .001; 11–20%: *F*(2, 50.98) = 5.972, *p* = .005] (Fig 4). Although not statistically significant, increases in postural lean angle for both strategies (ankle and torso) tended to increase GM activation during the remainder of the stance phase.

**Table 2:** Normalized stance phase EMG_RMS_ for each postural lean condition expressed as a percentage of the respective muscle’s peak activation while running upright (no lean) (N = 16).

		Postural Lean			
		Moderate		Large	
Muscle Activation (%)	No Lean	Ankle	Torso	Ankle	Torso
Gluteus Maximus	36.3 ± 11.8	41.1 ± 14.7	40.0 ± 12.5	49.1 ± 15.4	44.2 ± 15.6^†^
Biceps Femoris	29.1 ± 15.9	36.0 ± 19.6	36.4 ± 14.6	37.1 ± 15.9	37.3 ± 15.6^†^
Vastus Medialis	46.8 ± 18.3	40.7 ± 14.6	51.2 ± 13.8	41.8 ± 13.6	50.0 ± 20.3
Rectus Femoris	45.2 ± 22.1	35.9 ± 11.0	39.2 ± 16.5	35.8 ± 11.9	40.2 ± 19.4
Tibialis Anterior	27.4 ± 15.7	31.1 ± 14.1	34.3 ± 17.7	25.6 ± 12.2	26.7 ± 12.6
Soleus	55.2 ± 22.5	55.7 ± 19.4	54.1 ± 28.6	54.7 ± 12.5	51.6 ± 24.6
Medial Gastrocnemius	54.2 ± 23.9	53.4 ± 27.5	52.8 ± 26.6	50.2 ± 15.7	54.8 ± 22.8

Data are presented as Mean ± SD. Dagger (^†^) indicates a significant effect of postural lean angle across all postural lean angle conditions and both lean strategy conditions (p < .01).

Similar to the effect on GM muscle activation, increasing postural lean angle significantly increased stance phase BF muscle activation by 28% between upright and the large lean angle condition *F*(2, 56) = 5.343, *p* = .008 (Table 2). Post hoc analysis further revealed that the increased BF activation was primarily due to a ~50% increase in BF muscle activation during the loading response (first 20% of the gait cycle) of the stance phase [0–10%: F(2, 52.572) = 6.078, p = .004; 11–20%: F(2, 56) = 3.616, p = .033] (Fig 4). In contrast to GM and BF muscle activation, we observed no significant change in stance phase RF, SOL, MG, TA, and VM muscle activation although the activity of these muscles tended to decrease (~4–14%) from the upright to large lean conditions (p>.05, Table 2).

Finally, there was no significant effect of postural lean angle or lean strategy on any muscle activation during the swing phase of the running gait cycle. Moreover, there was no significant interaction effect between lean angle and lean strategy on stance or swing phase muscle activation.

## Discussion

The purpose of this study was to evaluate the effect of forward postural lean on running economy, kinematics, and muscle activation. Contrary to our initial support of the null hypothesis, our results reveal that forward postural lean during running influences running economy, lower limb kinematics, and muscle activation patterns. Most notably, running with an increased forward postural lean worsened running economy up to 8%. Furthermore, the effect of forward lean on running economy was the same whether participants leaned from the ankle or from the torso.

In accordance with our running economy results, we observed that increased lean angle had a greater influence on peak joint flexion than lean strategy did. Specifically, when running with an increased forward lean, peak hip and knee flexion increased during stance phase by 7% and 2%; respectively. Although greater forward lean angles consistently increased peak knee and hip flexion across both lean strategies, strategy can influence the effect of lean angle on peak knee and hip joint kinematics. These results indicate that the magnitude of lean angle influences peak hip and knee flexion kinematics more than lean strategy, but when combining lean angles and strategies, kinematics can change. Prior literature suggests that increased flexion at the hip and knee during the stance phase, reduces the effective mechanical advantage at the hip and knee (increasing torque) which in turn requires increased extensor muscle force to counteract the vertical ground reaction force [28–30]; this may be a reason we see a decrease in running economy.

As our angular kinematic results suggest, increased forward postural lean resulting in greater hip and knee flexion, likely reduces EMA which also possibly explains why hip extensor (biceps femoris and gluteus maximus) muscle activation increased during the stance phase of running while activation of other leg muscles remained unchanged. This was particularly true during the energetically costly collision portion of the stance phase (first 5–15% of stance) [31]. While not assessed in our study, Biewener et al., [28] observed that greater lower limb joint flexion decreases muscle EMA and increases the total active muscle volume, which contributes to a decreased running economy. A similar study to ours by Warrener et al., [24], found that running with greater trunk flexion increased the vertical impact transient peak ground reaction force and rate of loading, which are in agreement with our results, indicating that decreases in leg extensor muscle EMA during the collision and loading portion of stance may contribute to a reduced running economy. Warrener et al., [24] also observed hip extensor torque increased with increased lean. Hence, ours and Warrener’s results suggest that running with an increased forward lean increases hip joint torques, leading to greater hip extensor muscle activation to maintain dynamic postures and propel the body forward, which may help explain the reduced running economy. Additionally, the greater activation of gluteus maximus could reduce running economy in part because proximal lower limb muscles such as the gluteus maximus are hypothesized to generate force less efficiently than more distal muscles such as the ankle plantar flexors [32–34].

According to the spring-mass model of running and cost of generating force hypothesis, running economy is related to stride length and contact time [16, 35–37]. In our study, spatio-temporal kinematics including both stride length and contact time remained the same across the magnitude of lean angles. Contrary to our results, the recent study by Warrener et al., [24] saw that increased trunk flexion while running at 3.0 m/s notably decreased stride length, but increased stride frequency, and contact time. These differences in observed kinematics resulting from forward postural lean may be related to the much larger maximum forward trunk flexion angle (~ 36 degrees) used in Warrener et al.’s study. Nonetheless, it is interesting to note that even between their subjects’ preferred trunk flexion angle (9 degrees) and the moderate trunk flexion angle of 22 degrees, there was a 31% increase in the external hip moment. Such a large increase in external hip moment supports the hypothesis that running with increased forward postural lean reduces the EMA at the hip, increasing the external hip moment, and consequently increases the demand on the hip extensor muscles which may ultimately increase the metabolic cost of running.

### Limitations & future directions

A limitation of our study may be the specificity of participants tested. Due to the demographics of runners tested based on fitness requirements, some participants may have already developed a stride frequency close to optimal for the speed tested (3.58 m/s). As a result, experienced runners may be less prone to kinematic variability when compared to novice runners [38]; however, this limitation indicates there should be a decreased learning effect while running on a treadmill at this speed, which would increase the reliability of the results. Consequently, these findings may be less generalizable to a population of novice runners. Moreover, there are multiple strategies that subjects could use to achieve a particular lean angle within either the “ankle strategy” or “torso” strategy. Future research would benefit from different subject types and different lean angle variations. Additionally, although all subjects received the same directions to “try to lean from the ankle” or “try to lean from the torso” and used the same visual feedback system, running is accomplished as a multi-segment system, with many different neuromuscular possibilities, which could hypothetically meet our criteria with different biomechanical strategies.

For future research, we suggest controlling other running kinematic variables known to influence running economy such as foot strike pattern (heel strike or mid/forefoot). In addition, to better understand the influence of forward postural lean as a determinant of running economy and performance, future studies may want to explore using a wider range of forward lean angles or investigate the interaction between lean angle and slope of the ground.

Running takes place in a variety of unsteady environments, thus our understanding of running may benefit from future studies examining the effect of lean on the kinematics, muscle activation, and metabolic cost of running across a range of incline slopes, uneven surfaces and speeds. Another interesting area of exploration would be to understand how changes in lean angle while running affect the energetics, biomechanics, and neuromuscular strategies in fatigued participants.

Lastly, future research should use inverse dynamics and modeling techniques to further explore the hypothesis that forward postural lean while running decreases leg extensor EMA, increasing extensor muscle activation and leading to a higher metabolic cost of running.

## Conclusions

In summary, our results indicate that when running with a large forward postural lean, hip and knee flexion increased during stance, posterior chain muscle activation increased, while decreasing running economy. These findings suggest that running with a large forward lean may not be an effective postural strategy for running, particularly when the goal is to minimize the energetic cost over a given distance. Therefore, our results suggest that running with a more upright or moderate forward postural lean, either from the ankles or torso, may be more energetically optimal.

## Supporting information

S1 File (PDF)
