# Supplementary material for: The effect of forward postural lean on running economy, kinematics, and muscle activation
Source: PLoS One. 2024 May 29;19(5):e0302249. doi: 10.1371/journal.pone.0302249 (PMC11135760; doi:10.1371/journal.pone.0302249)
Supplement: S1 File — (PDF) [file pone.0302249.s001.pdf]

Subject_ID	Lean	A_vs_T	Condition	MASS	HEIGHT	AVE_ANKLE_STANCE	AVE_KNEE_STANCE	AVE_HIP_STANCE	AVE_PEAK_ANKLE_DORSIFLEX_STANCE	AVE_PEAK_ANKLE_PLANTFLEX_STANCE	AVE_PEAK_HIP_EXTENSION_STANCE	AVE_PEAK_HIP_FLEXION_STANCE	AVE_PEAK_KNEE_EXTENSION_STANCE	AVE_PEAK_KNEE_FLEXION_STANCE	AVE_STANCE_TIME_PER	AVE_STANCE_TIME_SEC	AVE_STEP_LENGTH_M	AVE_STEP_LENGTH_REL_HT	AVE_STRIDE_LENGTH_REL_HT	AVE_SWING_TIME_PER	AVE_SWING_TIME_SEC	AVE_TOTAL_LEAN	AVE_TOTAL_LEAN_MIDSTANCE	STRIDE_LENGTH_CV	STRIDE_WIDTH_CV
1	3	3	1	65.15	1.79	10.48812	-27.40346	1.65544	26.2137	-13.3029	-21.28758	21.65027	13.03245	39.98839	39.31459	0.25713	1.16622	0.65152	1.30373	60.68541	0.39687	4.07411	4.21920	0.01075	0.44113
2	3	3	1	74.75	1.84	13.78149	-24.66552	6.08409	29.14793	-12.03281	-20.49781	27.63526	8.02606	40.26754	44.7159	0.31757	1.26911	0.68974	1.3792	55.2841	0.3927	5.99079	5.88658	0.01198	0.10708
3	3	3	1	53.4	1.61	10.64211	-22.42409	10.78447	23.03599	-14.30495	-21.03595	32.14013	8.01109	37.27446	39.93741	0.25551	1.14392	0.71051	1.42097	60.06258	0.38426	4.15571	3.83912	0.01172	0.23378
4	3	3	1	68.2	1.75	4.58776	-13.11224	-3.17957	21.84434	-27.80791	-26.65599	15.49708	-1.70605	25.16183	31.07884	0.22	1.26389	0.72222	1.44464	68.92116	0.48795	4.96597	4.98343	0.01038	0.20407
5	3	3	1	62.5	1.685	6.48771	-28.53059	19.17969	18.14595	-13.98744	-2.38976	33.63654	16.95977	36.99861	28.08077	0.195	1.23905	0.73534	1.47074	71.91923	0.4995	5.97633	5.56769	0.01266	0.50441
6	3	3	1	57	1.66	6.34177	-25.84522	10.12174	23.89177	-30.90438	-22.88448	30.96104	6.49755	41.68349	39.3149	0.25405	1.15668	0.6968	1.39568	60.6851	0.3925	2.33705	1.18990	0.01035	0.13761
7	3	3	1	65.3	1.793	14.28578	-34.05795	22.24261	31.61938	-23.30428	-14.75242	43.68578	8.57193	51.84745	37.35759	0.28189	1.34587	0.75063	1.5017	62.64241	0.4727	5.99314	5.53037	0.01254	0.18938
8	3	3	1	56.8	1.723	13.89244	-30.63563	4.83612	32.06053	-17.71842	-20.233	21.8908	17.56345	42.53387	30.8095	0.22353	1.29306	0.75047	1.50125	69.19051	0.50205	6.16960	6.85049	0.01113	0.25937
9	3	3	1	58.6	1.74	8.78909	-26.20748	7.7527	25.35757	-23.10347	-20.7392	30.79369	9.50142	43.04873	39.50998	0.26798	1.20907	0.69487	1.38952	60.49002	0.4103	5.79072	6.28625	0.00841	0.16974
10	3	3	1	54.15	1.62	8.75077	-26.25542	8.50448	25.10388	-19.51077	-20.98707	37.90667	12.93834	41.46945	44.06747	0.27239	1.10383	0.68137	1.36241	55.93253	0.34571	4.43399	4.38931	0.01275	0.11225
11	3	3	1	46.1	1.59	6.69281	-27.16079	12.3452	22.74597	-20.7913	-17.97443	34.04475	10.40996	42.50254	41.29032	0.25539	1.10389	0.69427	1.38791	58.70968	0.36311	2.94605	2.52574	0.00789	0.17127
12	3	3	1	56.9	1.67	9.04576	-32.47593	9.82103	23.30344	-19.81656	-16.98801	31.2339	15.85052	45.55629	34.59377	0.22901	1.18167	0.70759	1.41459	65.40623	0.43298	6.39631	6.40700	0.0097	0.12875
13	3	3	1	57.8	1.67	8.99946	-28.43639	7.32423	25.48245	-21.50091	-24.17413	33.62287	5.5823	46.84079	43.02036	0.29848	1.2372	0.74084	1.48259	56.97964	0.3953	3.52854	3.61307	0.00813	0.15029
14	3	3	1	62.3	1.71	9.03282	-26.92198	16.37517	24.06379	-16.80428	-15.394	35.02458	7.88107	41.74667	35.43881	0.26466	1.33051	0.77807	1.55672	64.56119	0.48209	4.00225	4.16306	0.01198	0.19052
15	3	3	1	60.15	1.8	13.60795	-25.03423	17.49857	27.34673	-13.38718	-17.09002	35.69128	6.1636	40.93559	39.88959	0.30875	1.37951	0.7664	1.53301	60.11041	0.46524	5.19592	5.17837	0.00854	0.48608
16	3	3	1	73.25	1.91	15.59004	-38.16535	9.71497	29.39165	-13.66001	-15.38863	26.0782	20.0876	50.50185	31.14476	0.24074	1.37847	0.72171	1.4433	68.85523	0.53216	5.11041	4.90297	0.00897	0.32307
1	2	1	2	65.15	1.79	13.51697	-28.84671	10.58401	27.79831	-5.42673	-17.63288	27.79233	12.16433	42.72825	38.88798	0.25609	1.17457	0.65618	1.31331	61.11202	0.40241	8.24038	8.09966	0.01214	0.34177
2	2	1	2	74.75	1.84	16.13247	-28.48824	13.48719	30.80038	-8.5625	-16.76672	35.8247	10.81909	44.68938	45.90616	0.32528	1.26374	0.68681	1.37414	54.09383	0.38333	7.40334	7.20051	0.01206	0.18845
3	2	1	2	53.4	1.61	12.90412	-24.40105	17.00305	22.54636	-3.95985	-14.32094	35.53421	10.74009	37.05222	36.91041	0.23609	1.14047	0.70837	1.41612	63.08959	0.40348	8.08388	8.37397	0.01605	0.24274
4	2	1	2	68.2	1.75	5.48101	-12.89	12.35414	22.79868	-26.97447	-16.31044	31.53318	-6.78991	25.77913	31.83493	0.22872	1.28022	0.73156	1.46446	68.16507	0.48974	11.21225	11.30312	0.01833	0.28025
5	2	1	2	62.5	1.685	6.06543	-27.97495	25.22504	17.22148	-12.50522	3.34147	39.76656	15.7245	36.88251	28.68863	0.20075	1.24872	0.74108	1.4822	71.31137	0.499	8.30858	7.93892	0.01046	0.28176
6	2	1	2	57	1.66	5.08451	-24.35911	15.41308	21.89717	-31.11803	-19.13094	35.77952	4.22734	40.60137	40.6751	0.26893	1.17742	0.70929	1.42141	59.32491	0.39226	8.40208	8.26644	0.01297	0.15304
7	2	1	2	65.3	1.793	15.14886	-34.70448	28.76393	32.56472	-20.4112	-9.20135	50.14325	6.09359	53.52789	34.59236	0.27358	1.40882	0.78574	1.57223	65.40764	0.51726	9.30189	9.38136	0.01179	0.26791
8	2	1	2	56.8	1.723	16.08018	-29.86039	16.99214	34.50761	-18.16841	-13.39493	35.93204	8.77046	43.67207	31.64232	0.23158	1.30469	0.75722	1.51524	68.35768	0.5002	10.5337	10.59548	0.01136	0.35179
9	2	1	2	58.6	1.74	8.19748	-26.05198	11.87518	23.7365	-20.1407	-17.17639	34.02132	9.40723	42.14425	38.27901	0.25943	1.21047	0.69567	1.39012	61.72099	0.41836	7.01055	7.17202	0.01211	0.13676
10	2	1	2	54.15	1.62	8.4779	-25.284	15.33427	24.72449	-21.72333	-17.26418	41.9509	8.27405	41.74464	40.85854	0.26352	1.15055	0.71022	1.42086	59.14146	0.38148	6.90905	7.05854	0.01078	0.1981
11	2	1	2	46.1	1.59	6.77915	-27.27698	13.07093	22.00938	-18.68373	-15.65288	34.88925	11.02668	42.09796	39.34871	0.24039	1.08845	0.68456	1.36914	60.6513	0.3705	6.07782	6.0809	0.00814	0.20464
12	2	1	2	56.9	1.67	7.0971	-30.72291	20.56082	20.87095	-16.00961	-8.20948	41.30702	15.02065	44.85699	38.04075	0.24406	1.14425	0.68518	1.37067	61.95925	0.3975	9.38608	9.67385	0.01667	0.18386
13	2	1	2	57.8	1.67	8.43663	-29.53237	13.11894	25.00982	-23.55875	-21.09361	36.96928	5.14011	48.73532	41.72618	0.29718	1.27091	0.76103	1.52252	58.27382	0.41503	7.18293	7.21974	0.01004	0.09474
14	2	1	2	62.3	1.71	9.39839	-27.66603	25.11731	23.29354	-15.2035	-8.66162	41.65146	4.27927	42.58352	33.04952	0.24878	1.34219	0.78491	1.56979	66.95048	0.50392	6.74813	6.48203	0.01209	0.21041
15	2	1	2	60.15	1.8	12.75391	-21.39521	26.03548	26.23615	-14.4586	-8.64099	42.29007	2.42765	37.04955	39.96126	0.29605	1.32252	0.73473	1.46889	60.03874	0.44474	7.42518	6.89661	0.00903	0.20476
16	2	1	2	73.25	1.91	16.53808	-40.68011	21.6094	28.05808	-6.90509	-1.5677	36.77667	23.64011	51.03972	26.95701	0.2077	1.37545	0.72013	1.44024	73.04298	0.56277	9.5759	9.27577	0.01404	0.56964
1	1	1	3	65.15	1.79	14.56257	-23.68819	7.816	26.7063	-5.77625	-14.48336	21.92214	9.80054	35.07987	38.42377	0.25104	1.16562	0.65118	1.3026	61.57623	0.40232	4.01878	4.54244	0.01334	0.35262
2	1	1	3	74.75	1.84	14.97174	-26.49041	8.53079	30.33184	-8.66404	-17.99643	31.22161	10.26234	42.08369	45.12057	0.31769	1.25659	0.68293	1.36625	54.87943	0.38641	3.60145	3.66543	0.0149	0.17162
3	1	1	3	53.4	1.61	13.21508	-24.95705	16.42627	22.05226	-0.40976	-12.88535	33.48705	12.1817	36.54551	34.6987	0.21872	1.124	0.69814	1.39692	65.30129	0.41154	4.23427	4.95025	0.01725	0.21328
4	1	1	3	68.2	1.75	3.96338	-12.38861	0.09618	20.86053	-29.50083	-25.48645	19.68608	-5.16959	26.02403	30.68197	0.22105	1.28695	0.7354	1.47142	69.31802	0.49947	4.41236	4.80707	0.0085	0.26656
5	1	1	3	62.5	1.685	5.90907	-28.76358	24.28879	17.10459	-13.5172	3.03067	39.35742	17.11597	37.53832	28.61372	0.201	1.25406	0.74425	1.48854	71.38628	0.5015	4.43729	4.15664	0.01184	0.47059
6	1	1	3	57	1.66	4.73706	-25.27427	7.92209	20.96255	-30.89889	-23.33334	28.42146	8.94317	40.06335	39.89392	0.25698	1.15044	0.69304	1.3856	60.10609	0.38721	2.46227	2.41292	0.0178	0.15066
7	1	1	3	65.3	1.793	10.17004	-31.11452	16.72306	28.57478	-26.46349	-14.19165	39.93339	14.00106	47.97691	43.35406	0.2828	1.16549	0.65002	1.29688	56.64594	0.36936	5.06013	4.72659	0.01593	0.15522
8	1	1	3	56.8	1.723	12.69179	-27.03939	5.30094	29.5049	-18.65019	-19.62689	21.20828	13.49779	38.72953	28.53442	0.20699	1.29496	0.75157	1.50311	71.46558	0.5184	4.36722	4.75462	0.00956	0.38594
9	1	1	3	58.6	1.74	7.77294	-26.10573	7.38388	24.61154	-24.33395	-19.88958	31.53957	11.24028	42.68438	40.79134	0.27134	1.18855	0.68307	1.36459	59.20866	0.39384	3.32459	3.38676	0.00947	0.13
10	1	1	3	54.15	1.62	7.26881	-25.7839	8.14454	26.34979	-23.98798	-20.65387	37.4375	12.42096	40.896	43.79543	0.28295	1.15229	0.71129	1.42388	56.20457	0.36307	5.29826	5.83653	0.00952	0.15241
11	1	1	3	46.1	1.59	5.44874	-26.22689	10.87917	21.92153	-21.21459	-17.20659	32.79123	9.66407	41.05434	39.56174	0.23771	1.07142	0.67385	1.34751	60.43826	0.36314	4.05087	3.632	0.0066	0.13654
12	1	1	3	56.9	1.67	7.52955	-29.13922	15.01954	22.09637	-19.31483	-14.52423	36.4988	12.48875	44.15007	38.03569	0.24551	1.15229	0.68999	1.37991	61.96431	0.39994	4.48186	4.61522	0.01078	0.10061
13	1	1	3	57.8	1.67	8.53968	-30.27287	7.85589	26.19459	-24.47217	-23.59446	34.85532	6.3597	49.21085	44.44803	0.31006	1.24622	0.74624	1.49129	55.55197	0.3875	3.86585	4.11924	0.00801	0.12751
14	1	1	3	62.3	1.71	9.13989	-27.6018	20.02704	23.22095	-17.69313	-12.68467	38.3402	5.58766	42.11077	32.91516	0.24514	1.32738	0.77624	1.55423	67.08483	0.49973	4.27708	3.86059	0.00732	0.18475
15	1	1	3	60.15	1.8	12.43933	-24.09771	13.80256	26.15625	-15.01418	-19.33491	32.28863	4.76416	39.69291	38.89089	0.28559	1.30975	0.72764	1.45498	61.10911	0.44875	3.64742	3.5616	0.01273	0.63125
16	1	1	3	73.25	1.91	13.47687	-35.85157	8.94826	27.48681	-17.74446	-13.01965	26.24797	19.46601	47.3539	28.26225	0.21206	1.33777	0.7004	1.40104	71.73775	0.53824	4.79582	4.95238	0.00999	0.6084
1	2	2	4	65.15	1.79	11.06174	-21.47981	7.61168	24.1778	-6.03737	-15.5057	26.74199	6.14652	33.29052	41.9651	0.2651	1.12737	0.62982	1.25899	58.0349	0.36652	10.02789	10.46429	0.01457	0.39732
2	2	2	4	74.75	1.84	16.65417	-28.82932	11.99884	31.35596	-6.60781	-17.12334	34.30467	10.42561	44.83717	46.6478	0.32919	1.25881	0.68414	1.36815	53.3522	0.37649	7.29337	7.00213	0.01292	0.17099
3	2	2	4	53.4	1.61	15.64026	-24.88517	18.31337	27.839	-0.26435	-9.3319	34.05943	11.73159	36.29721	33.58245	0.21482	1.14054	0.70841	1.41622	66.41754	0.42495	8.96205	8.71087	0.01742	0.18839
4	2	2	4	68.2	1.75	4.45555	-13.04414	1.41505	20.67985	-26.95561	-22.64916	21.91224	-1.73076	25.29756	32.95466	0.22775	1.23233	0.70419	1.40895	67.04534	0.4635	10.9254	11.51128	0.01818	0.19691
5	2	2	4	62.5	1.685	4.9524	-28.74645	22.77864	17.0418	-15.07355	2.76404	39.46927	16.46317	37.57745	32.16566	0.22171	1.2282	0.7289	1.45803	67.83434	0.46756	9.3047	9.52415	0.01539	0.37981
6	2	2	4	57	1.66	6.61966	-26.08121	13.91367	20.594	-24.65917	-17.22155	34.23949	7.60501	39.62192	36.77616	0.24149	1.17101	0.70543	1.41035	63.22384	0.41512	8.9373	8.45491	0.01275	0.1788
7	2	2	4	65.3	1.793	12.6002	-32.2849	29.25951	29.53197	-20.81669	-5.45875	49.79645	7.1413	48.99749	35.06132	0.25044	1.27493	0.71106	1.42212	64.93868	0.46381	10.16486	10.7794	0.0181	0.36653
8	2	2	4	56.8	1.723	18.24105	-27.88281	5.32019	35.7616	-20.43534	-22.64161	23.97022	10.62917	40.20457	31.54824	0.22643	1.2821	0.74411	1.48833	68.45176	0.49129	8.61462	8.74022	0.00648	0.32056
9	2	2	4	58.6	1.74	9.50167	-27.19486	9.16811	24.12213	-19.73072	-19.35658	31.76579	10.51209	42.54345	37.08112	0.24988	1.20082	0.69013	1.38072	62.91888	0.42393	7.85658	7.85799	0.00772	0.13784
10	2	2	4	54.15	1.62	8.41163	-25.78029	12.68885	24.82411	-21.26434	-19.51674	38.90935	10.76717	41.79158	41.92811	0.26955	1.1454	0.70704	1.41476	58.07189	0.3733	6.29646	6.50432	0.0098	0.1478
11	2	2	4	46.1	1.59	5.03984	-26.49409	11.64616	22.57563	-22.52681	-17.23726	33.2719	10.07866	41.89577	40.38251	0.2437	1.07613	0.67681	1.35391	59.61749	0.35978	4.50589	4.4828	0.00638	0.12136
12	2	2	4	56.9	1.67	7.75927	-29.47792	25.61349	21.42734	-14.65945	-5.85563	46.93211	12.97969	44.71806	38.87345	0.2517	1.15661	0.69258	1.38465	61.12655	0.39574	8.74197	8.96584	0.01614	0.13587
13	2	2	4	57.8	1.67	9.96174	-30.23512	11.79775	26.1434	-18.85167	-23.08906	35.99406	6.07211	49.60167	42.37186	0.30122	1.26627	0.75825	1.51769	57.62814	0.40966	6.9588	7.12218	0.00873	0.11394
14	2	2	4	62.3	1.71	10.41521	-29.28601	23.51721	23.3658	-9.60891	-7.87753	41.37944	10.17319	43.14126	34.22686	0.25152	1.31163	0.76703	1.53421	65.77314	0.48331	6.47743	6.38614	0.00871	0.18769
15	2	2	4	60.15	1.8	12.05747	-22.86008	26.83467	24.69685	-11.89651	-4.01911	43.23801	3.19104	37.79987	42.73556	0.30224	1.26038	0.70021	1.40213	57.26444	0.405	7.31649	7.17813	0.01517	0.24832
16	2	2	4	73.25	1.91	10.94044	-35.12169	13.11395	26.38302	-17.85897	-8.72716	30.6546	20.52683	47.12514	30.91959	0.22858	1.32041	0.69132	1.38243	69.08041	0.51068	9.65804	10.02249	0.00976	0.52366
1	1	2	5	65.15	1.79	10.36572	-27.00319	-0.28745	26.61642	-12.8542	-23.38305	18.4663	10.04181	39.44963	42.30598	0.27366	1.15494	0.64522	1.29018	57.69402	0.37323	5.27772	5.60243	0.00958	0.39936
2	1	2	5	74.75	1.84	15.67424	-28.16143	9.37804	29.91693	-8.48869	-17.84327	31.07416	11.18186	42.73814	44.60117	0.31	1.23928	0.67352	1.34721	55.39883	0.38513	3.39004	3.21699	0.01326	0.15482
3	1	2	5	53.4	1.61	13.14344	-25.40839	17.65221	22.06491	0.06613	-11.59447	34.11808	12.44284	36.97848	35.04966	0.22048	1.12236	0.69712	1.39329	64.95034	0.40857	4.07273	4.99933	0.01461	0.17614
4	1	2	5	68.2	1.75	4.22462	-13.8222	-2.19653	20.73659	-28.20749	-25.59178	18.58737	-1.34162	26.37681	32.31218	0.2265	1.25059	0.71462	1.4295	67.68782	0.4745	4.02895	4.49305	0.01465	0.16147
5	1	2	5	62.5	1.685	7.03885	-28.69957	21.58584	17.92926	-13.72012	0.27829	38.0382	16.44492	38.17757	32.02249	0.21878	1.21676	0.72211	1.44324	67.97751	0.46463	4.51648	4.29776	0.01653	0.3617
6	1	2	5	57	1.66	5.5119	-25.60553	8.57446	20.91494	-27.25662	-22.13251	28.50504	9.62786	39.75679	39.81447	0.25314	1.1332	0.68265	1.36628	60.18553	0.38264	2.02696	1.75674	0.01362	0.13341
7	1	2	5	65.3	1.793	16.8224	-36.26015	30.26402	30.21826	-8.19985	-2.7198	49.03534	15.46718	50.77672	34.74939	0.24966	1.28024	0.71402	1.42949	65.25061	0.46916	4.14878	4.48634	0.01542	0.24329
8	1	2	5	56.8	1.723	17.87404	-31.53546	9.5324	34.8074	-16.21361	-18.47215	27.88758	14.13947	44.01653	30.64637	0.22703	1.32169	0.76709	1.53396	69.35363	0.51378	4.80257	4.73976	0.01007	0.28141
9	1	2	5	58.6	1.74	9.00146	-26.18035	10.00364	24.28196	-21.62346	-19.30146	32.911	9.92339	42.40543	39.41387	0.26509	1.19937	0.68929	1.37796	60.58614	0.40747	3.05046	3.27736	0.01048	0.11701
10	1	2	5	54.15	1.62	8.27052	-25.56814	9.20273	25.36344	-23.15451	-21.30642	36.79988	10.60335	40.64557	41.33696	0.26733	1.15391	0.71229	1.42357	58.66304	0.37939	5.24123	5.50808	0.01254	0.17483
11	1	2	5	46.1	1.59	4.72625	-26.32695	11.16548	22.58279	-24.02987	-17.00063	33.59262	9.89318	41.90485	41.05648	0.24848	1.07876	0.67847	1.35708	58.94352	0.35668	4.49299	4.23285	0.00807	0.11356
12	1	2	5	56.9	1.67	5.50343	-28.60798	15.5013	22.01343	-23.77209	-13.25406	37.68531	11.0323	44.43203	37.81666	0.25045	1.17943	0.70625	1.41399	62.18334	0.41176	4.21666	4.69619	0.01371	0.18316
13	1	2	5	57.8	1.67	10.08857	-30.51693	11.735	25.97675	-17.6383	-21.93324	36.10005	5.9529	49.23513	42.06401	0.2955	1.25312	0.75037	1.50058	57.93599	0.407	4.81038	4.57463	0.00817	0.10205
14	1	2	5	62.3	1.71	9.65721	-28.17698	19.71449	23.74118	-14.21596	-12.14693	38.31383	9.16736	42.43691	35.50198	0.25859	1.29628	0.75806	1.51779	64.49802	0.46981	5.10666	5.24797	0.00715	0.15041
15	1	2	5	60.15	1.8	12.82833	-25.58818	18.38971	26.3213	-13.23837	-13.64984	38.26427	7.29223	40.75839	43.06984	0.30859	1.27775	0.70986	1.42017	56.93016	0.40782	3.66166	3.56522	0.01197	0.25836
16	1	2	5	73.25	1.91	13.96341	-37.4043	13.51424	27.69952	-15.05324	-9.35821	30.08895	22.04771	48.63445	30.02801	0.22276	1.32474	0.69358	1.38687	69.97198	0.51904	4.09910	4.66749	0.00998	0.43998
1	0	1	6	65.15	1.79	9.77658	-22.43259	-1.46503	24.59508	-12.17621	-23.54384	19.00682	7.77926	34.59992	42.09496	0.27185	1.15142	0.64325	1.28703	57.90504	0.37372	1.35613	1.31731	0.01514	0.35367
2	0	1	6	74.75	1.84	14.75145	-27.2138	5.19602	30.45375	-12.69749	-20.90204	27.86191	10.86949	42.01397	43.97555	0.31395	1.27555	0.69323	1.38645	56.02445	0.4	1.51143	1.52729	0.01224	0.19053
3	0	1	6	53.4	1.61	11.84468	-25.15376	14.9962	22.20763	-6.17751	-14.39262	32.35722	12.34374	36.65558	34.80185	0.2205	1.12939	0.70148	1.40287	65.19814	0.413	2.02753	1.84844	0.01758	0.21584
4	0	1	6	68.2	1.75	3.85938	-12.84582	-4.8235	21.078	-28.48142	-28.20302	13.90413	-3.30132	26.25398	31.02077	0.22	1.2676	0.72434	1.44812	68.97923	0.48925	1.28147	1.39425	0.00917	0.21831
5	0	1	6	62.5	1.685	5.13233	-26.65544	18.29384	17.94794	-16.79875	-3.94442	34.96575	14.78519	36.76931	33.4325	0.2295	1.22443	0.72666	1.4537	66.5675	0.457	1.34925	1.48045	0.01525	0.33467
6	0	1	6	57	1.66	5.57363	-26.1646	6.86011	21.19171	-28.02402	-24.16649	26.00383	9.86386	40.01259	38.25173	0.24587	1.14703	0.69098	1.38333	61.74827	0.3969	1.51810	1.23837	0.01298	0.1079
7	0	1	6	65.3	1.793	16.4494	-36.2115	16.11895	29.62731	-13.85705	-13.69445	36.65257	16.79917	49.08839	34.17636	0.23017	1.20102	0.66984	1.34001	65.82364	0.44331	2.81048	2.67629	0.01092	0.29681
8	0	1	6	56.8	1.723	15.22375	-28.42104	2.01815	32.85821	-23.8711	-22.61669	21.01343	12.57061	40.48827	30.62393	0.21697	1.2622	0.73256	1.46465	69.37607	0.49151	1.88647	1.66212	0.00799	0.35387
9	0	1	6	58.6	1.74	8.60373	-25.98264	5.99976	24.35158	-23.77258	-22.12059	28.80809	10.74741	42.16053	38.95911	0.2631	1.20791	0.6942	1.38823	61.04089	0.41226	1.19001	1.34385	0.0103	0.09824
10	0	1	6	54.15	1.62	8.81122	-25.56237	6.46248	24.86353	-21.65457	-22.41659	33.46465	11.29532	40.60894	40.47179	0.25811	1.13812	0.70254	1.40543	59.52821	0.37961	3.52982	3.6172	0.00842	0.1834
11	0	1	6	46.1	1.59	8.32671	-27.62069	13.4967	22.50097	-10.73152	-15.65606	32.9627	11.88465	41.1647	37.77089	0.22745	1.07502	0.67612	1.35165	62.22911	0.37468	1.59294	1.59189	0.00882	0.22765
12	0	1	6	56.9	1.67	3.2025	-27.66881	5.59751	20.79545	-26.13593	-19.08921	28.96576	12.48136	41.77094	39.1041	0.25494	1.16242	0.69606	1.39222	60.8959	0.39699	1.17389	1.11453	0.0112	0.12821
13	0	1	6	57.8	1.67	9.05392	-29.78911	3.26566	26.28202	-25.24443	-25.9821	30.36738	7.48812	47.80086	42.19714	0.29402	1.24295	0.74428	1.48832	57.80286	0.4028	1.87425	2.02596	0.00662	0.14752
14	0	1	6	62.3	1.71	9.67391	-27.9844	13.78811	24.10286	-18.15608	-15.95406	33.72437	8.85336	41.84561	34.3881	0.24913	1.29056	0.75471	1.5097	65.6119	0.47531	2.20547	2.41928	0.00972	0.15313
15	0	1	6	60.15	1.8	14.06291	-26.11691	13.22813	28.3962	-12.3964	-20.12794	34.12947	8.96271	41.30849	43.29302	0.31353	1.29186	0.7177	1.4349	56.70698	0.41058	2.28914	2.07263	0.00859	0.3339
16	0	1	6	73.25	1.91	14.3538	-35.4006	6.95499	27.97983	-15.36	-16.74988	22.81065	17.73779	47.18844	28.74266	0.2173	1.34751	0.7055	1.41154	71.25734	0.53858	1.40317	1.32691	0.01264	0.42171
1	0	2	6	65.15	1.79	9.77658	-22.43259	-1.46503	24.59508	-12.17621	-23.54384	19.00682	7.77926	34.59992	42.09496	0.27185	1.15142	0.64325	1.28703	57.90504	0.37372	4.56139	4.51731	0.01514	0.35367
2	0	2	6	74.75	1.84	14.75145	-27.2138	5.19602	30.45375	-12.69749	-20.90204	27.86191	10.86949	42.01397	43.97555	0.31395	1.27555	0.69323	1.38645	56.02445	0.4	1.51143	1.52729	0.01224	0.19053
3	0	2	6	53.4	1.61	11.84468	-25.15376	14.9962	22.20763	-6.17751	-14.39262	32.35722	12.34374	36.65558	34.80185	0.2205	1.12939	0.70148	1.40287	65.19814	0.413	2.02753	1.84844	0.01758	0.21584
4	0	2	6	68.2	1.75	3.85938	-12.84582	-4.8235	21.078	-28.48142	-28.20302	13.90413	-3.30132	26.25398	31.02077	0.22	1.2676	0.72434	1.44812	68.97923	0.48925	1.28147	1.39425	0.00917	0.21831
5	0	2	6	62.5	1.685	5.13233	-26.65544	18.29384	17.94794	-16.79875	-3.94442	34.96575	14.78519	36.76931	33.4325	0.2295	1.22443	0.72666	1.4537	66.5675	0.457	1.34925	1.48045	0.01525	0.33467
6	0	2	6	57	1.66	5.57363	-26.1646	6.86011	21.19171	-28.02402	-24.16649	26.00383	9.86386	40.01259	38.25173	0.24587	1.14703	0.69098	1.38333	61.74827	0.3969	1.51816	1.23837	0.01298	0.1079
7	0	2	6	65.3	1.793	16.4494	-36.2115	16.11895	29.62731	-13.85705	-13.69445	36.65257	16.79917	49.08839	34.17636	0.23017	1.20102	0.66984	1.34001	65.82364	0.44331	2.81048	2.67629	0.01092	0.29681
8	0	2	6	56.8	1.723	15.22375	-28.42104	2.01815	32.85821	-23.8711	-22.61669	21.01343	12.57061	40.48827	30.62393	0.21697	1.2622	0.73256	1.46465	69.37607	0.49151	1.88647	1.66212	0.00799	0.35387
9	0	2	6	58.6	1.74	8.60373	-25.98264	5.99976	24.35158	-23.77258	-22.12059	28.80809	10.74741	42.16053	38.95911	0.2631	1.20791	0.6942	1.38823	61.04089	0.41226	4.19001	4.34385	0.0103	0.09824
10	0	2	6	54.15	1.62	8.81122	-25.56237	6.46248	24.86353	-21.65457	-22.41659	33.46465	11.29532	40.60894	40.47179	0.25811	1.13812	0.70254	1.40543	59.52821	0.37961	3.52982	3.6172	0.00842	0.1834
11	0	2	6	46.1	1.59	8.32671	-27.62069	13.4967	22.50097	-10.73152	-15.65606	32.9627	11.88465	41.1647	37.77089	0.22745	1.07502	0.67612	1.35165	62.22911	0.37468	1.59294	1.59189	0.00882	0.22765
12	0	2	6	56.9	1.67	3.2025	-27.66881	5.59751	20.79545	-26.13593	-19.08921	28.96576	12.48136	41.77094	39.1041	0.25494	1.16242	0.69606	1.39222	60.8959	0.39699	4.47389	5.21453	0.0112	0.12821
13	0	2	6	57.8	1.67	9.05392	-29.78911	3.26566	26.28202	-25.24443	-25.9821	30.36738	7.48812	47.80086	42.19714	0.29402	1.24295	0.74428	1.48832	57.80286	0.4028	3.87425	4.02596	0.00662	0.14752
14	0	2	6	62.3	1.71	9.67391	-27.9844	13.78811	24.10286	-18.15608	-15.95406	33.72437	8.85336	41.84561	34.3881	0.24913	1.29056	0.75471	1.5097	65.6119	0.47531	4.20532	4.41928	0.00972	0.15313
15	0	2	6	60.15	1.8	14.06291	-26.11691	13.22813	28.3962	-12.3964	-20.12794	34.12947	8.96271	41.30849	43.29302	0.31353	1.29186	0.7177	1.4349	56.70698	0.41058	2.28914	2.07263	0.00859	0.3339
16	0	2	6	73.25	1.91	14.3538	-35.4006	6.95499	27.97983	-15.36	-16.74988	22.81065	17.73779	47.18844	28.74266	0.2173	1.34751	0.7055	1.41154	71.25734	0.53858	5.4031	5.32691	0.01264	0.42171

Subject	Lean	A_vs_T	Condition	MASS_KG	HEIGHT_M	GROSS_COST	NET_COST
1	N/A	N/A	0	65.15	1.79	1.71	
2	N/A	N/A	0	74.75	1.84	1.08	
3	N/A	N/A	0	53.4	1.61	1.50	
4	N/A	N/A	0	68.2	1.75	1.26	
5	N/A	N/A	0	62.5	1.685	1.78	
6	N/A	N/A	0	57	1.66	1.26	
7	N/A	N/A	0	65.3	1.793	1.21	
8	N/A	N/A	0	56.8	1.723	1.43	
9	N/A	N/A	0	58.6	1.74	1.20	
10	N/A	N/A	0	54.15	1.62	0.44	
11	N/A	N/A	0	46.1	1.59	1.54	
12	N/A	N/A	0	56.9	1.67	1.45	
13	N/A	N/A	0	57.8	1.67	1.29	
14	N/A	N/A	0	62.3	1.71	1.16	
15	N/A	N/A	0	60.15	1.8	1.37	
16	N/A	N/A	0	73.25	1.91	1.65	
1	3	3	1	65.15	1.79	14.58	12.87
2	3	3	1	74.75	1.84	11.17	10.09
3	3	3	1	53.4	1.61	13.00	11.49
4	3	3	1	68.2	1.75	13.12	11.87
5	3	3	1	62.5	1.685	14.14	12.36
6	3	3	1	57	1.66	14.28	13.02
7	3	3	1	65.3	1.793	10.10	8.89
8	3	3	1	56.8	1.723	13.05	11.62
9	3	3	1	58.6	1.74	12.47	11.26
10	3	3	1	54.15	1.62	10.36	9.92
11	3	3	1	46.1	1.59	12.38	10.84
12	3	3	1	56.9	1.67	13.45	11.99
13	3	3	1	57.8	1.67	11.93	10.64
14	3	3	1	62.3	1.71	12.79	11.63
15	3	3	1	60.15	1.8	13.03	11.65
16	3	3	1	73.25	1.91	14.03	12.39
1	2	1	2	65.15	1.79	15.34	13.63
2	2	1	2	74.75	1.84	11.59	10.52
3	2	1	2	53.4	1.61	13.75	12.24
4	2	1	2	68.2	1.75	15.73	14.48
5	2	1	2	62.5	1.685	15.37	13.59
6	2	1	2	57	1.66	14.66	13.40
7	2	1	2	65.3	1.793	11.20	9.98
8	2	1	2	56.8	1.723	13.72	12.29
9	2	1	2	58.6	1.74	13.48	12.27
10	2	1	2	54.15	1.62	11.66	11.22
11	2	1	2	46.1	1.59	12.76	11.21
12	2	1	2	56.9	1.67	15.49	14.03
13	2	1	2	57.8	1.67	12.38	11.10
14	2	1	2	62.3	1.71	12.98	11.82
15	2	1	2	60.15	1.8	15.20	13.82
16	2	1	2	73.25	1.91	15.54	13.90
1	1	1	3	65.15	1.79	14.00	12.29

2	1	1	3	74.75	1.84	10.78	9.70
3	1	1	3	53.4	1.61	12.69	11.19
4	1	1	3	68.2	1.75	13.95	12.70
5	1	1	3	62.5	1.685	14.78	13.00
6	1	1	3	57	1.66	14.12	12.86
7	1	1	3	65.3	1.793	10.96	9.75
8	1	1	3	56.8	1.723	13.26	11.82
9	1	1	3	58.6	1.74	12.80	11.59
10	1	1	3	54.15	1.62	10.62	10.18
11	1	1	3	46.1	1.59	12.61	11.07
12	1	1	3	56.9	1.67	13.67	12.21
13	1	1	3	57.8	1.67	12.01	10.72
14	1	1	3	62.3	1.71	12.61	11.46
15	1	1	3	60.15	1.8	14.25	12.88
16	1	1	3	73.25	1.91	14.25	12.60
1	2	2	4	65.15	1.79	16.20	14.49
2	2	2	4	74.75	1.84	11.49	10.41
3	2	2	4	53.4	1.61	13.57	12.07
4	2	2	4	68.2	1.75	15.47	14.22
5	2	2	4	62.5	1.685	15.73	13.95
6	2	2	4	57	1.66	14.75	13.49
7	2	2	4	65.3	1.793	11.84	10.63
8	2	2	4	56.8	1.723	12.85	11.42
9	2	2	4	58.6	1.74	12.83	11.62
10	2	2	4	54.15	1.62	10.97	10.54
11	2	2	4	46.1	1.59	12.65	11.10
12	2	2	4	56.9	1.67	15.49	14.04
13	2	2	4	57.8	1.67	11.97	10.69
14	2	2	4	62.3	1.71	12.89	11.73
15	2	2	4	60.15	1.8	15.46	14.08
16	2	2	4	73.25	1.91	15.34	13.70
1	1	2	5	65.15	1.79	14.20	12.49
2	1	2	5	74.75	1.84	10.70	9.62
3	1	2	5	53.4	1.61	13.44	11.94
4	1	2	5	68.2	1.75	13.93	12.68
5	1	2	5	62.5	1.685	14.70	12.92
6	1	2	5	57	1.66	13.98	12.72
7	1	2	5	65.3	1.793	12.11	10.90
8	1	2	5	56.8	1.723	13.14	11.71
9	1	2	5	58.6	1.74	12.78	11.58
10	1	2	5	54.15	1.62	11.07	10.64
11	1	2	5	46.1	1.59	12.54	11.00
12	1	2	5	56.9	1.67	13.94	12.49
13	1	2	5	57.8	1.67	11.95	10.67
14	1	2	5	62.3	1.71	12.48	11.32
15	1	2	5	60.15	1.8	14.08	12.71
16	1	2	5	73.25	1.91	14.66	13.02
1	0	0	6	65.15	1.79	14.19	12.48
2	0	0	6	74.75	1.84	11.02	9.94
3	0	0	6	53.4	1.61	12.71	11.21
4	0	0	6	68.2	1.75	13.86	12.61

5	0	0	6	62.5	1.685	14.02	12.24
6	0	0	6	57	1.66	13.91	12.65
7	0	0	6	65.3	1.793	11.36	10.15
8	0	0	6	56.8	1.723	12.62	11.19
9	0	0	6	58.6	1.74	12.83	11.62
10	0	0	6	54.15	1.62	10.55	10.12
11	0	0	6	46.1	1.59	12.53	10.99
12	0	0	6	56.9	1.67	12.86	11.41
13	0	0	6	57.8	1.67	11.75	10.46
14	0	0	6	62.3	1.71	12.59	11.43
15	0	0	6	60.15	1.8	13.57	12.20
16	0	0	6	73.25	1.91	14.43	12.78
